# Methodological Quality of Economic Evaluations in Integrated Care: Evidence from a Systematic Review

**DOI:** 10.5334/ijic.4675

**Published:** 2019-09-09

**Authors:** Mudathira Kadu, Nieves Ehrenberg, Viktoria Stein, Apostolos Tsiachristas

**Affiliations:** 1 International Foundation for Integrated Care, Oxford, UK; 2 Institute of Health Policy, Management and Evaluation, University of Toronto, Toronto, Ontario, CA; 3 Health Economics Research Centre, Nuffield Department of Population Health, University of Oxford, Oxford, UK

**Keywords:** integrated care, economic evaluation, quality assessment, methodology, systematic review

## Abstract

**Introduction:**

The aim of this review is to systematically assess the methodological quality of economic evaluations in integrated care and to identify challenges with conducting such studies.

**Theory and methods:**

Searches of grey-literature and scientific papers were performed, from January 2000 to December 2018. A checklist was developed to assess the quality of economic evaluations. Authors’ statements of challenges encountered during their evaluations were qualitatively coded.

**Results:**

Forty-four articles were eligible for inclusion. The review found that study design, measurement of cost and outcomes, statistical analysis and presentation of data were the areas with most quality variation. Authors identified challenges mostly related to time horizon of the evaluation, inadequate or lack of comparator group, contamination bias, and a post-hoc evaluation culture.

**Discussion:**

Our review found significant differences in quality, with some studies showing poor methodological rigor; challenging conclusions on the cost-effectiveness of integrated care.

**Conclusion:**

It is essential for evaluators to use best-practice standards when planning and conducting economic evaluations, in order to build a reliable evidence base for decision-making in integrated care.

## Introduction

Health care systems around the world are seeking strategies that improve efficiency in the organization and delivery of care to meet increasing care needs and rising healthcare costs [1]. In the United States, failure to coordinate care; particularly for populations with chronic illness, cost the healthcare system about $35 billion to $45 billion in 2011 [2]. Integrated care is one of the most prominent strategies which have been proposed as a means to improve population health, organize complex care and control healthcare cost growth [1,3]. The recent paradigm shift in care delivery, from acute and segmented care, to preventive and coordinated care, is in alignment with the WHO Framework on Integrated People-centred Health Services. This framework calls for more integrated approaches to how services are funded, managed and delivered [4].

The process of integrating care is not straightforward and dependent on several factors such as availability of resources, health and social care systems, and commitment and support of stakeholders [1,4]. As a result, different types of integrated care models have been designed and implemented around the world. These differ by the type of population served, care sectors and services integrated, care providers involved, and health care financing and reimbursement structures set in place [1]. However, despite the increasing implementation of integrated care models, the scientific evidence on its cost-effectiveness remains ambivalent [5].

Economic evaluations examining whether integrated care interventions can achieve value for money, are becoming increasingly common alongside larger evaluation studies [6]. The economic evidence that has been summarized in systematic reviews and meta-analyses is generally inconclusive [8,9,10,11,12,13,14]. A review by De Bruin et al. reported that 62% of disease management programmes were cost saving [13]. A recent meta-analysis by Desmedt et al. found that 50% of integrated care interventions for multiple sclerosis, 67% for schizophrenia and 89% for diabetes were cost-effective [9]. Ouwens et al found that 57% of the studies on integrated care interventions were cost-saving [8]. There are many possible explanations for the inconclusiveness. This may include the widely varying definitions and components of integrated care programmes implemented and measures of impact chosen in the evaluation [5,7,8,15]. Yet, the most commonly suggested reasons are related to the methodological quality of the evaluations [5,15]. In their review of systematic reviews on this topic, Nolte and Pitchforth concluded that *“the quality of existing economic evaluations is the main impediment to arriving at a robust evidence base that is suitable to inform decision making in integrated care”* [5, p. 37]. Despite best practice recommendations [16,17], very few have conducted quality assessment of studies included in systematic reviews examining the impact of integrated care [9,12,13,18]. Little is known about current practices in conducting economic evaluation of integrated care interventions, or the methodological gaps related to the overall study designs, measurements of impact, analysis and presentation of results.

Results of economic evaluations are increasingly being used to inform healthcare decision-making, which further underscores the need to examine whether these evaluations meet methodological standards [19]. Policy makers concerned with the transferability of integrated care models across healthcare settings may be interested in knowing whether the conclusions expressed in studies are acceptable and trustworthy [20,21]. This is especially the case since allocating public funds to healthcare services informed by misleading or biased economic analysis, may pose significant ethical issues and policy implications [19,22]. Employing reliable methods in economic evaluations may also be of importance to clinicians and health practitioners considering integrated care in their health service planning and service delivery [20]. Finally, a systematic assessment of the quality of evidence generated by economic evaluation is important in contextualizing the substantial variation in findings of integrated care interventions, as well as in identifying opportunities for improvements [5]. Therefore, the aim of this review is to systematically assess the methodological quality of economic evaluations in integrated care and to identify challenges with conducting such studies.

## Methods

### Search strategy

Relevant search terms related to the broad concepts of “integrated care” and “economic evaluation” were identified by looking at frequently used terms in previous systematic reviews [5,7,9,10,12,14] and seminal literature [17,19,20,21,23,24] on the respective topics. Our search was also informed by terms used in the Integrated Care Search tool developed by the International Foundation for Integrated Care [25]. See Appendix 1 for search terms used in the review.

Searches of grey-literature and scientific papers were performed, from January 2000 to December 2018, in the following databases: Medline/PubMed, EMBASE, CINAHL, Web of Science, Scopus, the World Health Organization Library and Information Networks for Knowledge database (WHOLIS), Database of Abstracts of Reviews of Effects (DARE) and the NHS Economic Evaluation Database (NHS EED), and the OECD Library. In addition, hand searching of reference lists in key publications (including systematic reviews on integrated care, dissertations, conference proceedings, opinion pieces, editorials and conference abstracts) was used to identify any relevant missed articles.

### Selection process and eligibility criteria

Citations were downloaded and screened in Mendeley, an online citation manager tool. All article abstracts, and titles were read independently by two reviewers based on the inclusion criteria detailed below. If the reviewers could not determine whether to exclude an article based on its abstract and title, then it was retrieved for full text reading until agreement was reached.

#### Inclusion criteria

1. Articles published in English.
2. Articles that described the implementation, execution or evaluation of interventions or programmes based on Kodner and Spreeuweberg’s definition of integrated care: “funding, administrative, organisational, service delivery and clinical interventions designed to create connectivity, alignment and collaboration within and/or between the cure and care sectors” [26, p. 3].
3. Empirical economic evaluations as defined by Drummond et al.: “the comparative analysis, measurement, valuing and identification of alternative courses of actions in terms of their cost and consequences” [20, p. 3].

### Data abstraction and analysis

Two data abstraction templates were adapted from Boland et al. [12], and aligned with elements from the PICO framework for research [27]. The first template was used to extract information about the studies including: the study objectives, the settings, description of the intervention, target population and size. The table was also used to abstract information on the evaluation, including study design, the type of economic analysis, the evaluation perspective, the length of the observation period and the measures used.

The second template was developed to assess the quality of economic evaluations. This was in the form of a checklist appraising the economic evaluation methodology and the risk of bias in the study design of the interventions. It provided a binary scoring criterion (yes/no) assessing strengths and weakness of the studies on 30 items. The checklist was a combination of: 1) the Consolidated Health Economic Evaluation Reporting Standards (CHEERS) [17], and 2) the Health Technology Assessment of Disease Management Programmes (HTA-DM) [28]. The CHEERS guideline was developed by an expert panel to optimize the reporting of health economic evaluations [17], while the HTA-DM has been previously used to assess the overall quality of evaluations [28]. Two reviewers (MK, NE) abstracted the information from both templates, and then independently cross-verified each other’s information for reporting consistency and data reliability.

Our approach to identifying challenges and limitations with their overall evaluations was modeled after the thematic analysis approach used in a systematic review by Thomas and Harden [29]. This involved three stages: 1) coding text, 2) developing descriptive themes from lifted texts and 3) generating analytical themes. Firstly, in the coding phase, when articles described barriers, limitations or challenges that emerged from the economic evaluation of integrated care, they were regarded as “attributive statements” and systematically recorded. These statements were often found in the discussion and results section of the articles. In the second stage, descriptive themes that emerged from the lifted texts were identified. Finally, three reviewers (MK, NE and VS) used an inductive approach to identify the dominant themes that emerged from clustering statements of challenges [30].

## Results

**Figure 1:**
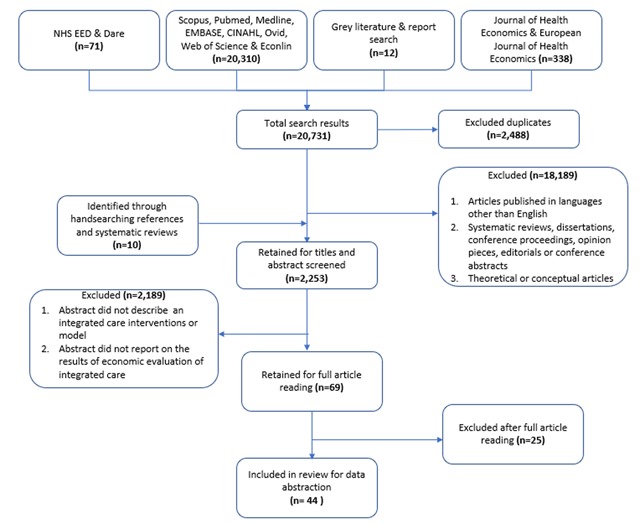
Flowchart of study inclusion at various stages of the selection process.

The initial literature search and screening based on the inclusion criteria yielded 2,263 abstracts. Most of these articles were rejected as they were not based on interventions that fulfilled the definition of integrated care and did not report the results of economic evaluations. After full text reading of 69 articles, 44 articles were eligible for inclusion. Figure 1 shows our PRISMA flowchart at various stages of the selection process.

The most common types of economic evaluations were cost-utility analysis [31,32,33,34,35,36,37,38,39,40,41,42,43,44,45,46,47,48,49], followed by cost-consequence [50,51,52,53,54,55,56,57,58,59,60,61,62,63], cost-effectiveness [64,65,66,67] and cost-comparison analysis [68,69,70,71,72]. For more information on the different types of economic evaluations, please see Drummond et al. [19]. Studies conducted economic evaluations in various regions, which were based in North America (n = 16), United Kingdom (n = 2), Europe (n = 20), Australia (n = 1), Africa (n = 3) and Asia (n = 2). The sample size of the studies varied from 36 individuals in the intervention group, to large-scale population-level evaluations of integrated care initiatives. The interventions targeted various groups including older adults with chronic illness or frailty, children, individuals with mental illness and/or substance abuse, individuals treated for multiple occurring chronic conditions or multimorbidity and high users of the healthcare system. Many interventions were delivered across sectors, more commonly integrating acute, primary and secondary care. Others were single-sited and focused on evaluating clinical integration at the multidisciplinary team level. Please refer to Table 1 for description of the studies, and patient and intervention characteristics.

**Table 1:** Description of study, patient and intervention characteristics.

Study characteristics								Patient characteristics				

Reference	Country	Study design	Type of economic evaluation	Pers-pective	Inter-vention Size	Control Size	Observation period	Target population	Intervention description	Setting	Study objective(s)	Measures

Zulman et al. (2017)	USA	RCT	Cost-consequence	Health care payer	150	433	17 months	High healthcare users (top 5%)	The ImPACT multidisciplinary team addressed health care needs and quality of life through comprehensive patient assessments, intensive case management, care coordination, and social and recreational services	Primary care medical home	To evaluate the impact of augmenting the Veterans Affairs’ medical home and multidisciplinary team with an intensive management program	Outcomes: 1) patient satisfaction, 2) patient activation measures; Cost: inpatient and outpatient services
Weisner et al. (2001)	USA	RCT	Cost-effectiveness	Health care payer	285	307	6 months	Adults with alcohol and drug dependence	Patients received treatment through an integrated model, in which primary health care was included within the addiction treatment program	Primary care within substance abuse program	Examine differences in treatment outcomes and costs between integrated and independent models of medical and substance abuse care	Outcomes: 1) alcohol and drug abstinence rate, 2) healthcare utilization; Cost: inpatient, outpatient and treatment costs
Weeks et al. (2009)	USA	Cross-sectional	Cost-comparison	Health care payer	63,647	677,901	NA	Individuals 65 years and older under Medicare	The intervention group was assignment to a large multispecialty group practice in accountable care organizations. Each beneficiary was assigned to a unique primary care physician for a 2-year period.	Multispecialty primary care group practice	Compare the costs and quality of care provided to Medicare beneficiaries by physicians who worked within large multispecialty physician group practices	Outcomes: 1) outpatient clinical measures, 2) ambulatory care-sensitive hospitalisations; Cost: inpatient, long-term and home care
Weaver et al. (2009)	USA	Cluster RCT	Cost-consequence	Health care payer	232	199	12 months	Individuals with HIV, mental illness and substance abuse disorders	Integrated HIV primary care, mental health, and substance abuse services among triply diagnosed patients.	Outpatient multidisciplinary mental health, substance abuse and case management services	Evaluate the cost-effectiveness of integrating HIV primary care, mental health, and substance abuse services amongst triply diagnosed patients	Outcomes: 1) quality of life, 2) mental health scores; Cost: 1) inpatient, outpatient, rehabilitation, home care, alternative, primary care, long-term care; 2) out-of-pocket expenses
Van Orden et al. (2009)	The Netherlands	Cluster RCT	Cost-consequence	Health care payer	102	63	12 months	Adults with mental illness	Patients with mental illness were assigned to collaborative care program in a primary care setting through traditional referral of patients to mental health services	Primary care and specialized mental health care	Compare the effect of introducing collaborative care on the attached mental health professional model in a primary care setting	Outcomes: 1) quality of life, 2) satisfaction with care, 3) mental health score; Costs: treatment costs
Olsson et al. (2009)	Sweden	Pre-post cohort	Cost-effectiveness	Health care payer	56	56	18 months	Community dwelling older adults 65 years and older, with hip fracture	Patient centered integrated care pathway for patients admitted with hip fracture.	Multidisciplinary orthopedic hospital ward	Compare costs and consequences of integrated care pathways for patients admitted with acute hip fractures	Outcomes: 1) activities of daily living score; Cost: 1) intervention and operational costs, 2) implementation costs, 3) inpatient costs
Leeuwen et al. (2015)	The Netherlands	Cluster RCT	Cost-utility	Societal perspective	456	691	24 months	Community dwelling older adults with frailty	The Geriatric Care Model combined regularly scheduled in-home comprehensive geriatric assessments by practice nurses followed by a customized care plan management and training of practice nurses by a regional geriatric expert team	Multidisciplinary geriatric primary care team	Evaluate the cost-effectiveness of the Geriatric Care Model compared to usual primary care	Outcomes: 1) quality adjusted life years, 2) activities of daily living; Costs:1) inpatient, primary, outpatient, home and long-term care & medication costs, 2) informal care giver costs
Lanzeta et al. (2016)	Spain	Cluster RCT	Cost-utility	Health care payer	70	70	12 months	Individuals with multimorbidity	An integrated healthcare model comprising an assigned internist and a hospital liaison nurse for patients with multimorbidity	Primary and hospital-based care	Examine the effectiveness of an integrated model for patients with multimorbidity, based on an assigned internist and a hospital liaison nurse	Outcomes: 1) quality adjusted life years, 2) health resource utilization; Costs: 1) acute, specialists, primary and home care, 2) treatment costs
Goorden et al. (2013)	The Netherlands	RCT	Cost-utility	Societal perspective	65	61	12 months	Employees sick-listed due to major depressive disorder	Collaborative care for major depressive disorder in an occupational healthcare setting	Occupational health setting and consulting specialist care	Evaluate the cost-utility of a collaborative care intervention in sick-listed employees with major depressive disorder	Outcomes: 1) quality adjusted life years, 2) health care utilization; Costs: 1) primary, specialist care and intervention costs, 2) productivity loss
Boland et al. (2015)	The Netherlands	Cluster RCT	Cost-utility	Societal perspective	554	532	24 months	Patients with chronic obstructive pulmonary disease	A multidisciplinary team was trained in motivational interviewing, setting up individual care plans, exacerbation management, implementing clinical guidelines and redesigning the care process	Multidisciplinary primary care teams	Examine the cost-effectiveness of a disease management program for patients living with chronic obstructive pulmonary disease	Outcomes: 1) quality adjusted life years, 2) symptom improvement; Costs: 1) acute, primary, rehabilitation and home care, 2) productivity loss and travel costs
Donohue et al. (2014)	USA	RCT	Cost-utility	Health care payer	150	152	12 months	Patients with depression following coronary artery bypass graft (CABG) surgery	Patients who screened positive for depression after CABG surgery received an 8-month centralized, nurse-provided and telephone-delivered CC intervention for depression	Primary care and specialized outpatient mental health care	Examine the impact of telephone-delivered collaborative care of treating post-CABG surgery depression compared to usual care	Outcomes: 1) quality adjusted life years, 2) depression free days; Costs: acute and outpatient costs
Cohen et al. (2012)	Canada	Pre-post cohort	Cost-consequence	Societal perspective	81	Self-comparator	12 months	Children with medically complex chronic conditions	Clinics at two community hospitals distant from tertiary care were staffed by local community pediatricians with the tertiary care center nurse practitioner and linked with primary care providers	Outpatient clinics within community-based hospitals with pediatricians, linked with primary care	Evaluate the effectiveness of a community–based complex care clinic integrated with a tertiary care facility	Outcomes: 1) health related quality of life; 2) perceptions of care; Cost; 1) inpatient, primary, outpatient and home care. 2) Out-of-pocket expenses for health and social care
Wise et al. (2006)	USA	Cohort	Cost-comparison	Health care payer	2010	30,360	12 months	Older adults, 65 years and older, with chronic conditions	A prospective health risk assessment, point-of-care information management, clinical decision support, multidisciplinary clinical oversight, and a clinical “Health Navigator” to deliver integrated health care	Multidisciplinary primary care teams	Assess the impact of an integrated set of care coordination tools and chronic disease management interventions on utilization and cost	Cost: adjusted acute, primary care and drug costs
McCall et al. (2010)	USA	Cohort	Cost-consequence	Health care payer	2,619	2490	36 months	Older adults, aged 65 and older who are high cost Medicare users	The intervention was a practice-based care management services to high-cost Medicare beneficiaries. Case managers, who were assigned to each physician office, developed relationships with program participants to provide support across the continuum of care	Multidisciplinary colocated primary care teams with linkages to home and long-term care	Evaluate whether the Massachusetts General Hospital and its case management program can meet targeted cost-savings compared to control	Outcomes: 1) comorbidity score, 2) care experience and satisfaction; 3) healthcare utilization; Cost: 1) covered inpatient, primary, outpatient and home care
Simon et al. (2001)	USA	RCT	Cost-effectiveness	Health care payer	110	109	6 months	Primary care patients with major depressive episode	Stepped collaborative care for patients with persistent depressive symptoms after usual primary care management. Patients received collaborative care with liaison psychiatrist and primary care physician.	Large primary care clinics part of a health cooperative	Evaluate the incremental cost-effectiveness of stepped collaborative care for patients with persistent depressive symptoms after usual primary care management	Outcome: 1) depression-free days; Costs: outpatient, primary, specialists and inpatient care
Hebert et al. (2008)	Canada	Pre-post cohort (D-in-D)	Cost-consequence	Health care payer	501	419	48 months	Older adults aged 65 living with frailty and disability	Integrated Service Delivery System developed to improve continuity and increase the efficacy and efficiency of services, especially for older and disabled populations	Population level health and social care including: acute, home, long term, rehab and social services	Evaluate the impacts of integrated care model for older adults on the use of services and on costs in the experimental zone, compared with the comparison zone	Outcomes: 1) functional and mental health scores; 2) care satisfaction; 3) care giver burden; Costs: 1) implementation and operation costs, 2) primary, specialist, acute and outpatient costs
Vroomen et al. (2012)	The Netherlands	Cluster RCT	Cost-utility	Societal perspective	201	136	6 months	Older adults living in residential homes	The intervention consisted of quarterly in-home assessment of residents, multidisciplinary team meetings with primary care physicians, nurse and physiotherapists, and multidisciplinary consultations	multidisciplinary residential home care linked with primary care	Evaluate the cost-effectiveness of a multidisciplinary integrated care in residential homes	Outcomes: 1) quality adjusted life years, 2) functional status, 3) quality of care scores; Costs: 1) acute, primary, outpatient/specialist care, 2) operational/implementation costs; 3) informal caregiver productivity loss
Salmon et al. (2012)	USA	Pre-post cohort	Cost-comparison	Health care payer	39,982	Self-comparator	12 months	Patients enrolled in collaborative accountable primary care organizations	A collaborative accountable care model with registered nurses who served as care coordinators were a central feature of the initiative. They used patient-specific reports and practice performance reports to improve care coordination, identify gaps, and address opportunities for quality improvement	primary care physician group practice	Examine the impact of accountable coordinated care initiative in three diverse provider practices before and after implementation	Outcomes: 1) Outpatient/primary care clinical measures; Cost: 1) Inpatient and primary care, 2) intervention cost
Looman et al. (2016)	The Netherlands	Pre-post cohort	Cost-utility	Societal perspective	254	249	12 months	Community dwelling older adults with frailty	Primary care physician served as the care coordinator and single-entry point for the elderly. Nurse practitioner visited patients for cognitive, mental and functional assessment who also provided case management. A multidisciplinary treatment plan was then developed.	Multidisciplinary primary care team linked with nursing home and outpatient/specialist care	Examine the impact of integrated model for community-dwelling older adults with frailty	Outcome: 1) quality adjusted life years; Cost: 1) inpatient, primary, home, outpatient and nursing home care, 2) intervention operational costs, 3) Informal care giver costs
Celano et al. (2016)	USA	RCT	Cost-utility	Health care payer	92	91	6 months	Patients hospitalized for cardiovascular illness with mental illness	Psychiatric treatment in the intervention was provided in concert with the patients’ primary medical clinicians—within a framework supervised by a psychiatrist	Inpatient care followed by with telephone outpatient follow up, with primary care linkages	Examine the cost-effectiveness and differences in healthcare utilization and cost between collaborative depression and anxiety program with usual care	Outcomes: 1) quality adjusted life years, 2) mental health status; Costs: 1) acute, primary, outpatient/specialist care
Markle-Reid et al. (2010)	Canada	RCT	Cost-consequence	Societal perspective	55	54	6 months	Community dwelling older adults, 75 years and older, at risk for falls	A six-month multifactorial and evidence based falls prevention strategy involving a multidisciplinary team	Multidiscplinary home care linked with primary care and community services	Determine the effects and costs of a multifactorial, multidisciplinary team approach to falls prevention compared with usual home care services.	Outcomes: 1) falls; 2) clinical outcomes (functional, mental and cognitive scores); 3) quality adjusted life years; Cost: 1) Acute, home, primary and community care, 2) out-of-pocket indirect medical expenses
Pozzilli et al. (2002)	Italy	RCT	Cost-consequence	Health care payer	133	68	12 months	Patients diagnosed with multiple sclerosis	The home-based multidisciplinary team collaborated with the patient, physician, and caregiver in designing individualized clinical care and in coordinating home services with hospital care	Multidisciplinary home care with specialists linkages	Compare the effectiveness and the costs of multidisciplinary home-based care in multiple sclerosis with hospital care	Outcomes: 1) quality of Life, 2) health resource utilization; Costs: 1) inpatient, outpatient and home care, 2) intervention costs
Tzeng et al. (2007)	China	RCT	Cost-consequence	Health care payer	257	247	6 months	Individuals diagnosed with schizophrenia	A network of mental health services was created by coordinating a general acute care hospital, a day hospital, a psychiatric rehabilitation institution, a community rehabilitation center, home visit providers, a specialized psychiatric hospital, and local clinics	Network of acute care, day hospital, rehabilitation, home care providers and local clinics	Compare the cost-effectiveness of an integrated model of schizophrenia treatment with those of the traditional treatment model provided by acute care	Outcomes: 1) quality of life, 2) care giver burden, 3) health service utilization, Costs: inpatient, outpatient, rehabilitation and home care
Bergmann et al. (2017)	Malawi and Mozambique	Pre-post cohort (D-in-D)	Cost-effectiveness	Health care payer	Not reported	Not reported	24 months	Children under 5 years with HIV who were underweight	Integration of health and nutrition program areas identified as important in reducing the vulnerability of children impacted and infected by HIV/AIDS: infant and young child feeding, prevention of mother-to-child transmission of HIV, pediatric HIV care and treatment, and community-based management of acute malnutrition	Community based health workers and community clinics for HIV and acute under-nutrition	To estimate the impact and cost-effectiveness for integrated HIV and nutrition service delivery in sub-Saharan Africa	Outcomes: 1) HIV infections averted, 2) undernutrition cases cured, 3) disability adjusted life years; Cost: 1) intervention costs, 2) operational/implementation costs, 3) life long HIV treatment cost
Koch et al. (2017)	USA	Pre-post cohort (D-in-D)	Cost-comparison	Health care payer	2.5 million per year	2.5 million per year (their own control)	15 months	Patients served by hospital and physician groups merged as part of horizontal integration of care	Vertical integration through a set of physician acquisitions by hospital systems	Hospital, primary and specialist care physicians	Assess how (financial) vertical integration affects volume and cost of services provided by acquired physicians and hospitals	Outcome: health care utilization; Cost: acute, primary and outpatient/specialist care
Rosenheck et al. (2016)	USA	Cluster RCT	Cost-utility	Health care payer	223	181	24 months	Individuals aged 15-40 in treatment for first episode of psychosis	A multidisciplinary, team-based treatment approach for first episode psychosis. This included: personalized medication management, family psychoeducation, individual, resilience-focused illness self-management therapy, and supported education and employment	Multidisciplinary community mental health treatment clinics	Compare the cost-effectiveness of a comprehensive, multidisciplinary, team-based treatment approach for first episode psychosis to usual community care	Outcomes: 1) quality adjusted life years, 2) health service utilization; Costs: 1) inpatient, outpatient, residential and nursing home care and medication costs, 2) implementation and operational costs
Sahlen et al. (2016)	Sweden	RCT	Cost-utility	Health care payer	36	36	6 months	Patients diagnosed with congestive heart failure	The patients were offered structured person-centered palliative care at home with easy access to care, and the team was responsible for the total care, including co-morbidities	Multidisciplinary palliative home care team linked with specialist care	To assess the cost-effectiveness of person-centered integrated heart failure and palliative home care	Outcomes: 1) quality adjusted life years; Costs: 1) acute, home, primary and specialist care, 2) intervention cost
Blom et al. (2016)	Netherlands	RCT	Cost-consequence	Societal perspective	3145	4133	12 months	Community-dwelling older adults, 75 and older living with complexity	The general practitioner (GP) or the practice nurse (under supervision of the GP) made an integrated care plan for participants with complex problems. Other care professionals were involved where needed through multidisciplinary consultations	General Practice with geriatric assessment training	Assess the effectiveness and cost- effectiveness of a monitoring system to detect the deterioration in somatic, functional, mental or social health	Outcomes: 1) quality of life, 2) activities of daily living, 3) satisfaction with care 4) Informal care giver time; Cost: 1) acute, primary, outpatient, nursing home and medication 2) intervention costs, 3) implementation costs, 4) informal care costs
Pimperl et al. (2017)	Germany	Pre-post cohort	Cost-consequence	Health care payer	5411	5411	48 months	Individuals enrolled with the accountable care organization insurance scheme	Accountable care organization focused on population health management with a Triple Aim framework	Cross-sectoral cooperation of physicians, hospitals, social care, nursing staff, therapists, and pharmacies	Identify an appropriate study design for evaluating population health outcomes of accountable care organization such as based on shared savings contract	Outcomes: 1) survival, 2) comorbidity score; 3) Costs: outpatient physician and specialist care, hospital, rehabilitation, medication costs
Schellenberg et al. (2004)	Tanzania	Cohort	Cost-consequence	Societal perspective	100,000	100,000	24 months	Children with malaria, pneumonia, malnutrition and diarrhea	The intervention was designed to increase children’s survival at household, community, and referral levels, with three components: improvements in case-management, improvements in health systems, and improvements in family and community practices	Family and community primary care practices and hospitals	Assess the effectiveness of facility- based integrated management of childhood illness in rural Tanzania	Outcomes: 1) child health outcomes, 2) household health behavior 3) children’s mortality; Costs: 1) drugs and vaccines, 2) implementation costs & operational costs, 3) intervention costs, 4) out-of-pocket expenses, 5) acute, primary and community care costs
Bird et al. (2012)	Australia	Pre-post cohort	Cost-consequence	Health care payer	223	Self-comparator	36 months	Children with asthma that presented frequently at the emergency department	Patients received care facilitators who provided assistance in the promotion of carer/self-management, education and linkage to an integrated healthcare system, comprising of acute and community-based healthcare providers.	Acute, primary and other community-based care	Assess a model of care for pediatric asthma patients aimed to promote health and reduce their acute care utilizations	Outcomes: 1) activity limitation and emotional function, 2) acute care utilizations; Cost: 1) intervention costs; 2) acute care costs
Goltz et al. (2013)	Germany	Cohort	Cost-consequence	Health care payer	2455	2455	36 months	Patients with osteoporosis who experienced index fractures	Patients received multidisciplinary cooperation between different sectors of the health care system, improved diagnostics, optimized drug therapy as well as encouraging lifestyle changes such as adequate nutrition and exercise.	Ambulatory care	Evaluate the outcomes of patients participating in a program of integrated care for osteoporosis in terms of medication supply, fracture incidence and expenses	Outcomes: 1) fracture Incidence, 2) occurrence of pain; Costs: 1) acute care costs, 2) treatment costs, 3) medication costs
Steuten et al. (2007)	Netherlands	pre-post cohort	Cost-utility	Societal perspective	2455	2455	60 months	Patients 18 years and older with GP diagnosis of asthma	Care was delivered by a collaborative practice team consisting of a pulmonologist, primary care physician, and respiratory nurse specialists. The respiratory nurse specialists act as liaison between primary and secondary care	primary care collaborating with specialists	Assess long-term cost-utility of a disease management program for adults with asthma was assessed compared to usual care	Outcomes: 1) quality adjusted life years, 2) asthma related exacerbations/control; costs: 1) acute, primary and outpatient/specialist care costs, 2) medication costs 3) treatment costs, 4) implementation and operational costs, 5) patient productivity loss
Wiley-Exley et al. (2009)	USA	Cluster RCT	Cost-utility	Societal perspective	1257	1948	6 months	Older adults, 65 years and older with major depressive disorder in primary care	Patients required referral to a behavioral health provider outside the primary care setting, and the behavioral health provider had primary responsibility for the mental health/substance abuse needs of the patient	Multidisciplinary specialist team colocated in primary care	Compare the cost-effectiveness of integrated care in primary care to enhanced specialty referral for older adults with behavioral health disorders	Outcomes: 1) depression free days, 2) quality adjusted life years; Costs: 1) inpatient, emergency room use, nursing home, rehabilitation care. 2) medication costs, 3) caregiver and patient indirect costs (transportation, productivity loss)
Karow et al. (2012)	Germany	Cohort	Cost-utility	Health care payer	64	56	12 months	Adults patients diagnosed with first or multiple-episode schizophrenia	Patients receive a multidisciplinary team with a small client/staff ratio, home-treatment, high-frequent treatment contacts, no dropout policy and 24-hour availability	Inpatient, outpatient/specialists and occupational therapy care	To compare the cost effectiveness of therapeutic assertive community treatment with standard care in schizophrenia.	Outcomes: 1) quality adjusted life years, Costs: 1) inpatient care, day-clinic care, outpatient and specialist care costs, 2) medication costs
Renaud et al. (2009)	Burundi	Cohort	Cost-effectiveness	Health care payer	149	Self-comparator	60 months	People living with HIV who initiated antiretroviral treatment	Care was given in primary health care facilities, which favours a more personal link with patients. Secondly, these health facilities delivered integrated care for people living with HIV.	Primary care based on non-for-profit organization delivering care for individuals living with HIV	Calculate the incremental cost effectiveness of an integrated care package for people living with HIV/AIDS in a not-for-profit primary health care centre.	Outcomes: 1) disability adjusted life years; Cost: 1) outpatient, acute and home care costs 2) medication costs 3) intervention costs, 4) food support costs
Tanajewski et al. (2015)	United Kingdom	RCT	Cost-utility	Health care payer	205	212	3 months	Older people at risk of adverse outcomes after acute care discharge	This intervention comprised geriatrician assessment of patients on the acute medical unit and further short-term community follow-up to continue the assessment and oversight of the delivery of medical and non-medical community interventions	Multidisciplinary acute care team and links to primary care	To examine the cost-effectiveness of a specialist geriatric medical intervention for frail older people in the 90 days following discharge from an acute medical unit	Outcomes: 1) quality adjusted life years; Costs: 1) acute, primary and specialist care; 2) intervention costs
Lambeek et al. (2010)	Netherlands	RCT	Cost-utility	Health care payer	66	68	12 months	Individuals visiting outpatient clinic due to low back pain	Integrated care consisted of a workplace intervention based on participatory ergonomics, with involvement of a supervisor, and a graded activity programme based on cognitive behavioural principles	Occupational health setting linked with multidisciplinary outpatient team	To evaluate the cost effectiveness of an integrated occupational health programme for sick listed patients with chronic low back pain	Outcomes: 1) duration until sustainable return to work; 2) quality adjusted life years; Costs: 1) primary and secondary care, home care, and drugs. 2) Out of pocket expenses for additional and informal care; 3) patient productivity loss
Bertelsen et al. (2017)	Denmark	RCT	Cost-utility	Societal perspective	106	106	12 months	Adult patients admitted to the hospital with acute coronary syndrome	A model of shared care cardiac rehabilitation that included general practitioners and the municipality	Shared care between primary care and outpatient public health centers, with multidisciplinary teams	To assess the cost-utility of shared care cardiac rehabilitation versus hospital-only cardiac rehabilitation from a societal perspective	Outcomes: 1) quality-adjusted life years; Costs: 1) intervention cost/formal and informal staff time; 2) primary and secondary care; 3) productivity loss
Camacho et al. (2018)	United Kingdom	Cluster RCT	Cost-utility	Health care payer	191	196	24 months	Patients with depressive symptoms and a record of diabetes and/or coronary heart disease	Participants attending primary care physician practices allocated to the collaborative care group received up to eight face-to-face sessions of brief psychological therapy delivered by a case manager over 3 months	General practices with case managers co-located with multidisciplinary team	To assess the cost-effectiveness of collaborative care for people with depression in the context of multimorbidity	Outcomes: 1) depression severity; 2) quality adjusted life years; 3) health care utilization; Costs: 1) inpatient; 2) outpatient; 3) emergency; 4) primary/community care; 5) intervention costs; 6) Implementation and training costs
Everink et al. (2018)	The Netherlands	Cohort	Cost-utility	Societal perspective	113	49	9 months	Community-dwelling older patients who were admitted to a geriatric rehabilitation facility	The integrated care pathway comprised of cross-organizational agreements on coordination and continuity of care for older patients who transfer between the hospital, the geriatric rehabilitation facility and primary aftercare in the home context	Coordination between the hospital, the geriatric rehabilitation and primary care and home care	To determine the cost-effectiveness of receiving usual care compared to receiving care in the integrated care pathway	Outcomes: 1) dependence in activities of daily living; 2) quality adjusted life years; Cost: 1) intervention costs; 2) implementation costs; 3) primary, home care, long-term care, acute care and allied professionals; 4) patient out-of pocket expenses; 5) informal caregiving
Wong et al. (2018)	China	RCT	Cost-utility	Health care payer	43	41	24 months	End-stage heart failure patients referred to in-hospital palliative care services	Patients received a transitional homebased palliative end-stage heart failure program delivered by nurse case managers who were trained specialist palliative care home care nurses with experience in heart failure management	Transitional care between hospital to home care delivered by case manager and multidisciplinary home care team	To evaluate the cost-effectiveness of a transitional home-based palliative care program	Outcomes: 1) quality adjusted life years; Costs: 1) acute, home and emergency care; 2) intervention cost; 3) training cost
Uittenbroek et al. (2018)	The Netherlands	RCT	Cost-utility	Societal perspective	747	709	12 months	Older adults, aged 75 and over with primary care providers	A primary care physician-led Elderly Care Team was assembled for each participating practice, which also consisted of an elderly care physician, a community nurse, and a social worker	General practitioner-led elderly multidisciplinary care team in primary care with case manager	To assess the cost-effectiveness of integrated geriatric care team in primary care	Outcomes: 1) quality adjusted life years; 2) number of days older adult was able to age in place (i.e. no nursing home stays); Costs: 1) primary, acute, medication and paramedical care; 3) social and home care; 4) informal caregiving
Tsiachristas et al. (2015)	The Netherlands	Cohort	Cost-utility	Societal perspective	1034	1034	24 months	patients diagnosed with or at risk of cardiovascular disease and chronic obstructive pulmonary disorder	Programs focused on improving the collaboration between different disciplines of health care professionals and redesigning the care giving process toward proactive, patient-centered care.	Disease management programs implemented through collaborations between general practices and hospitals, primary care practices (including physiotherapists and dieticians), or primary and community settings	To evaluate the cost-effectiveness of disease management programs for patients diagnosed with or at risk of cardiovascular disease and chronic obstructive pulmonary disorder	Outcomes: 1) quality adjusted life years; 2) level of physical activity; 3) proportion of smokers; Costs: 1) health care utilization costs; 2) travel costs; 3) productivity loss; 4) development costs; 5) implementation costs

### Quality assessment

#### Study design

Randomized control trials (58%) were the most commonly used study designs, followed by observational cohort designs (41%), where 16% of the studies were quasi-experimental or used pre-post cohort designs. Only one study was cross-sectional in nature.

To minimize bias while conducting the economic evaluations, 91% of the studies identified a comparator or control group, which did not receive the intervention. However, the baseline population characteristics in the groups were significantly different in almost a third of the studies, particularly in observational cohort designs. Quasi-experimental approaches such as difference-in-difference analysis of the pre-post intervention period were conducted in 7% of the studies. This was performed to ensure that the evaluations were in fact measuring the impact of the intervention rather than other confounding factors that might bias the results. Authors such as Pimperl et al. used propensity-score matching to ensure individuals enrolled in an accountable care organization were similar at baseline to their comparator group on socio-demographic and clinical variables [63]. Majority of the longitudinal studies collected data at multiple points, spanning beyond baseline and follow-up (61%). Furthermore, 70% had clear description of patient attrition or drop-outs at the follow-up period. Contamination due to the exposure of individuals in the comparator to the intervention, occurred in a third of the studies. To avoid this bias, 20% of the studies used a cluster RCT design, where individuals were randomized at the care setting level, rather than at the patient level.

#### Measurement of cost and outcomes

Most studies explicitly stated the perspective adopted in their economic evaluation (75%), with the healthcare payer perspective dominating the broader societal perspective (66% vs. 34%). Studies that adopted a societal perspective also considered the indirect impact of the intervention on caregiver burden, out of pocket care expenses and productivity loss. Non-medical and indirect costs were considered in 35% of the studies. For example, van Leeuwen et al. valued the indirect costs of a multidisciplinary geriatric primary care team on the informal care of frail elderly patients [31]. Only a third of the studies included costs associated with the development of the intervention or the implementation costs, with the majority only considering intervention and healthcare utilization costs. Healthcare costs and utilization from across all relevant health and social sectors were reflected in 66% of the studies. However, while some studies reflected both types of information, others only reported overall health care costs without the resource utilization. For example, in a cross-sectional study of a large multispecialty primary care group practice in the US serving Medicare recipients, despite including all relevant health care costs (such as home healthcare, long-term care, skilled nursing facilities and acute care), the resource utilization associated with each sector was not reported [68]. The follow-up period varied across studies, from short time horizons such as 3 months [34], to up to 5 years [49,67]. Those that measured the impact of the interventions within a year did not require discounting of costs and health benefits (53%) because they were expressed in present values [73]. However, only 18% of the studies with a time horizon longer than a year applied discounting.

#### Statistical analysis and presentation of data

Full economic evaluations of integrated care interventions applied the incremental cost-effectiveness ratio analysis (ICER) in their approach (55%), rather than using the net monetary (NMB) or health benefit (NHB) (7%). Studies that reported the ICER and NHB estimated the joint monetary and effect differences between the intervention and a comparator. Various approaches were adopted to address uncertainty around the reported cost-effectiveness estimates, including presenting both cost-effectiveness acceptability curves and cost-effectiveness planes. This involved demonstrating whether the integrated care intervention met or surpassed society’s willingness to pay (WTP) for an additional unit of health benefit (43%). For example, in an evaluation of a community-based intervention for frail older adults, Looman et al. graphically reported their ICERs on a cost-effectiveness plane [46]. Using this approach, they demonstrated that compared to usual care, the new intervention was only 0.21% less costly and more effective, while 78.8% more costly and less effective. Because decision-criteria such as WTP thresholds or cut-off points were not applied to the results, it was challenging to determine whether this intervention could be conclusively deemed cost-effective [46]. On the other hand, Tanajewski et al determined that a multidisciplinary discharge program was more favorable than usual care using the NMB analysis at various WTP thresholds (£20,000–120,000). They also showed that the more decision-makers were willing to invest in the intervention, the higher the probability of its cost-effectiveness [34]. Subgroup analysis was used to examine the heterogeneity of economic impact or the source of variability, but was conducted in 47% of the studies in this review for more information, please refer to Table 2 for the checklist assessing the quality of economic evaluations.

**Table 2:** Checklist assessing the quality of economic evaluations.

Category	Item Description	Zulman et al. (2017)	Weisner et al. (2001)	Weeks et al. (2009)	Weaver et al. (2009)	Van Orden et al. (2009)	Olsson et al. (2009)	Leeuwen et al. (2015)	Lanzeta et al. (2016)	Goorden et al. (2013)	Boland et al. (2015)	Donohue et al. (2014)	Cohen et al. (2012)	Wise et al. (2006)	McCall et al. (2010)	Simon et al. (2001)	Hebert et al. (2008)	Vroomen et al. (2012)	Salmon et al. (2012)	Looman et al. (2016)	Celano et al. (2016)	Markle-Reid et al. (2010)	Pozzilli et al. (2002)	Tzeng et al. (2007)	Bergmann et al. (2017)	Koch et al. (2017)	Rosenheck et al. (2016)	Sahlen et al. (2016)	Blom et al. (2016)	Pimperl et al. (2017)	Schellenberg et al. (2004)	Bird et al. (2012)	Goltz et al. (2013)	Steuten et al. (2007)	Wiley-Exley et al. (2009)	Karow et al. (2012)	Renaud et al. (2009)	Tanajewski et al. (2015)	Lambeek et al. (2010)	Bertelsen et al. (2017)	Camacho et al. (2018)	Everink et al. (2018)	Kam et al. (2018)	Ulttenbroek et al. (2018)	Tsiachristas et al. (2015)	%

**Study design**	1. Design was experimental (e.g. RCT or cluster-RCT) or quasi experimental design (e.g. used propensity score matching, pretest-posttest design)?	✓	✓	○	✓	✓	✓	✓	✓	✓	✓	✓	○	○	○	✓	✓	✓	○	✓	✓	✓	✓	✓	✓	✓	✓	✓	✓	✓	○	○	○	✓	✓	○	○	✓	✓	✓	✓	○	✓	✓	✓	75
	2. Random allocation into intervention and control groups	✓	✓	○	✓	✓	○	✓	✓	✓	✓	✓	○	○	○	✓	○	✓	○	○	✓	✓	✓	✓	○	○	✓	✓	✓	○	○	○	○	○	✓	○	○	✓	✓	✓	✓	○	✓	✓	○	57
	3. The study population consist of an intervention and control group	✓	✓	✓	✓	✓	✓	✓	✓	✓	✓	✓	○	✓	✓	✓	✓	✓	○	✓	✓	✓	✓	✓	✓	○	✓	✓	✓	Y	○	✓	✓	✓	✓	✓	✓	✓	✓	✓	✓	✓	✓	✓	✓	91
	4. Relevant baseline characteristics are comparable	✓	✓	○	✓	✓	✓	✓	✓	✓	✓	✓	○	○	✓	✓	○	✓	○	○	✓	✓	✓	✓	○	○	✓	NA	✓	✓	○	○	○	✓	✓	✓	○	✓	✓	✓	✓	✓	✓	✓	✓	72
	5. The interventions or strategies being compared are described	✓	✓	✓	✓	✓	✓	✓	✓	✓	✓	✓	✓	✓	✓	✓	✓	✓	✓	✓	✓	✓	○	✓	✓	✓	✓	✓	✓	✓	✓	✓	✓	✓	✓	✓	✓	✓	✓	✓	✓	✓	✓	✓	✓	98
	6. Included more than just baseline and follow up period	✓	○	○	✓	✓	○	✓	○	✓	✓	✓	✓	○	✓	✓	✓	✓	○	✓	✓	○	○	○	○	✓	✓	○	○	✓	○	○	✓	✓	✓	✓	○	○	✓	✓	✓	✓	✓	○	✓	61
	7. Clear description of inclusion and exclusion	✓	✓	✓	✓	✓	✓	✓	✓	✓	✓	✓	✓	○	✓	✓	✓	✓	○	✓	✓	✓	✓	○	○	✓	✓	✓	✓	✓	✓	✓	○	✓	✓	✓	○	✓	✓	✓	✓	✓	✓	✓	✓	86
	8. Clear description of drop-outs	✓	✓	○	✓	○	✓	✓	✓	○	✓	✓	○	○	✓	○	✓	✓	○	✓	○	✓	✓	✓	○	✓	✓	○	✓	✓	○	○	○	✓	✓	✓	✓	✓	✓	✓	✓	✓	✓	✓	✓	70
**Intervention setting**	9. Stated relevant aspects of the system(s) in which intervention takes place	✓	✓	✓	✓	✓	✓	✓	✓	✓	○	○	✓	✓	✓	✓	✓	✓	✓	✓	✓	✓	✓	✓	✓	✓	○	✓	✓	✓	✓	✓	✓	✓	○	✓	✓	✓	○	✓	✓	✓	○	○	✓	84
	10. Co-interventions or contamination are avoided	○	○	○	○	✓	○	✓	✓	○	✓	○	○	✓	○	○	○	✓	○	○	○	○	○	✓	○	○	✓	○	✓	○	✓	○	○	✓	✓	○	✓	○	○	○	✓	○	○	○	○	32
**Measurement of costs & outcomes**	11. Describe the perspective of the study and relate this to the outcomes and costs being evaluated.	○	○	○	✓	○	✓	✓	✓	✓	✓	✓	✓	○	○	✓	✓	✓	✓	✓	✓	✓	✓	○	○	○	✓	✓	✓	○	○	✓	✓	✓	✓	✓	✓	✓	✓	✓	✓	✓	✓	✓	✓	75
	12. Described which outcomes were used as the measure(s) of benefit in the evaluation	✓	✓	✓	✓	✓	✓	✓	✓	✓	✓	✓	✓	✓	✓	✓	✓	✓	✓	✓	✓	✓	✓	✓	✓	✓	✓	✓	✓	✓	✓	✓	✓	✓	✓	✓	✓	✓	✓	✓	✓	✓	✓	✓	✓	100
	13. Inclusion of development and implementation cost	○	○	○	○	○	✓	○	○	○	✓	○	○	○	○	○	✓	✓	○	○	○	○	○	○	✓	○	✓	○	✓	○	✓	○	○	✓	✓	○	○	○	○	✓	✓	✓	✓	○	✓	34
	14. Inclusion of cost & utilization from across all relevant health and social sectors	✓	✓	✓	✓	○	○	✓	✓	✓	✓	○	✓	○	✓	○	✓	✓	○	✓	○	✓	✓	✓	○	○	✓	○	✓	✓	✓	○	○	✓	✓	○	○	✓	✓	✓	✓	✓	○	✓	✓	66
	15. Inclusion of direct non-medical and indirect costs	○	○	○	○	○	○	✓	○	✓	✓	○	✓	○	○	○	✓	✓	○	✓	○	✓	○	○	○	○	○	○	✓	○	✓	○	○	✓	✓	○	○	○	✓	✓	○	✓	○	✓	✓	39
	16. Justification for omitting costs categories	○	○	○	✓	○	✓	○	○	✓	N/A	○	○	○	○	✓	○	✓	○	✓	NA	○	○	○	○	○	○	✓	NA	○	✓	○	○	NA	○	○	✓	○	○	NA	○	✓	✓	○	NA	29
	17. The sources of resource utilization and cost are described	✓	✓	✓	✓	○	✓	✓	✓	✓	✓	✓	✓	✓	✓	✓	✓	✓	○	✓	✓	✓	✓	○	✓	✓	✓	✓	✓	✓	✓	✓	✓	✓	✓	✓	✓	✓	✓	✓	✓	✓	○	✓	✓	91
	18. The resource utilization and costs are reported separately	✓	✓	✓	✓	✓	✓	○	✓	✓	✓	○	✓	○	✓	✓	✓	✓	○	○	✓	○	✓	✓	○	✓	✓	✓	✓	✓	○	✓	✓	○	○	✓	✓	✓	✓	✓	○	✓	✓	✓	✓	75
	19. Reports the (adjusted) dates of estimated resource quantities and unit costs	○	○	✓	✓	○	✓	✓	○	✓	✓	✓	✓	○	○	○	✓	✓	✓	✓	○	✓	✓	○	○	○	✓	✓	✓	✓	✓	○	✓	✓	✓	✓	✓	✓	✓	✓	Y	Y	Y	Y	Y	73
	20. Discounting of outcomes and costs performed	○	NA	○	NA	○	○	Y	NA	○	○	NA	NA	NA	○	○	○	Y	NA	NA	NA	NA	NA	NA	○	○	○	NA	NA	○	○	○	○	Y	NA	NA	Y	NA	N/A	NA	○	NA	NA	NA	○	18
**Statistical analysis**	21. Data analysis is performed according intention-to-treat principle	✓	○	○	✓	✓	○	✓	○	○	✓	○	○	○	○	○	✓	✓	○	○	○	○	✓	○	○	○	○	○	✓	○	○	○	○	✓	○	○	✓	✓	✓	✓	✓	○	✓	✓	○	39
	22. Dealt adequately with missing observations	✓	○	○	✓	✓	○	✓	○	✓	✓	✓	○	○	○	○	○	✓	○	✓	✓	✓	○	○	○	✓	○	✓	✓	✓	○	○	○	✓	✓	✓	✓	✓	✓	✓	✓	✓	○	✓	✓	59
	23. Appropriate statistical methods for analysing skewed data	○	✓	○	✓	○	✓	✓	✓	✓	✓	✓	○	○	○	✓	✓	✓	○	✓	✓	○	✓	○	○	✓	✓	✓	✓	○	○	✓	○	✓	✓	✓	○	✓	✓	✓	✓	✓	✓	✓	✓	68
	24. Report the values, ranges, references, and if used, probability distributions for all parameters.	✓	✓	✓	✓	✓	✓	✓	✓	✓	✓	✓	✓	○	✓	✓	✓	✓	○	✓	✓	✓	✓	✓	○	✓	✓	✓	✓	✓	✓	✓	✓	✓	✓	✓	○	✓	✓	✓	✓	✓	✓	✓	✓	91
	25. Analysed cost-effectiveness using the incremental cost- effectiveness ratio (ICER) method	○	✓	○	○	○	○	✓	✓	✓	✓	✓	○	○	○	✓	○	✓	○	✓	✓	○	○	○	✓	○	✓	○	○	○	○	○	○	✓	✓	✓	✓	✓	✓	✓	✓	✓	✓	✓	✓	55
	26. Analysed cost-effectiveness using the incremental net- monetary or health benefit (INB) regression method	○	○	○	○	○	○	○	○	○	○	○	○	○	○	○	○	○	○	○	○	○	○	○	○	○	✓	○	✓	○	○	○	○	○	○	○	○	✓	○	○	○	○	○	○	○	7
	27. Performed sub group analysis to examine heterogeneity of results	✓	✓	○	○	○	○	✓	✓	○	✓	○	○	✓	○	○	✓	✓	✓	○	○	✓	○	○	○	✓	✓	○	✓	○	○	✓	✓	✓	✓	○	○	✓	✓	○	○	○	○	✓	✓	48
	28. Analysed the uncertainty in the estimates of the costs and effects	✓	✓	✓	✓	○	○	✓	✓	✓	✓	✓	○	○	○	✓	○	✓	○	✓	✓	○	○	○	○	○	✓	✓	✓	○	○	○	○	✓	✓	✓	✓	✓	✓	✓	✓	✓	✓	✓	✓	64
**Presentation of data**	29. A decision criteria is applied to determine whether to reject or accept intervention (e.g. willingness-to-pay vs. cost effectiveness threshold)	○	✓	○	○	○	○	✓	✓	○	✓	✓	○	○	○	○	○	✓	○	○	✓	○	○	○	○	○	✓	○	✓	○	○	○	○	✓	✓	✓	○	✓	✓	✓	✓	✓	✓	✓	○	43
	30. The study discusses the generalizability of the results to other context and/or patient groups	✓	✓	✓	✓	✓	✓	✓	✓	✓	✓	✓	✓	✓	✓	✓	✓	✓	✓	✓	✓	✓	✓	✓	✓	✓	✓	✓	✓	✓	✓	✓	✓	✓	✓	✓	✓	✓	✓	✓	✓	✓	✓	✓	✓	98

Legend: yes ✓, no ○.

### Emerging Challenges

#### Time horizon

Limitation associated with the study timeframe was the most frequently cited barrier to conducting robust evaluations of integrated care interventions. In a cost-effectiveness analysis of a multidisciplinary residential care for frail older adults, Vroomen et al. note that: “the [six months] duration of the trial was relatively short because of the high risk for drop out owing to the extreme vulnerability of residents” [45, p. 5]. A further limitation of the study’s internal validity was the patients’ maturation effect: “patients in a residential home have a heterogeneous mix of chronic conditions that naturally erode over time which makes it difficult to know if an intervention would be able to override the downward trend…in such a short time span” [45, p. 6]. Other authors questioned whether measures such as mortality or quality adjusted life years (QALYs), often the standard in economic evaluations, were sensitive enough to truly capture the effect of the intervention in short follow-up periods [45,63].

Underestimation of the downstream health and monetary benefits of integrated care interventions was also a significant concern in studies with short time horizons [42]. In a 12-month cost-utility analysis of a collaborative care program targeting patients with depression and cardiovascular illness, Donohue et al. highlight that in this type of intervention, “most cost savings [occur] between the first and second years of follow-up, stressing the continued need for adequately powered and longer-term trials” [44, p. 457]. Zulman et al. argue that when determining an appropriate follow-up period, it is important to not only account for the implementation period but also the potential initial intensity of patients care needs: “once patients are enrolled in such programs, it takes time to build their trust, modify health behaviors and improve chronic disease trajectories….which could translate to increased [initial need for] health maintenance and screening, but subsequent reductions in future utilizations” [50, p. 172].

#### Finding suitable comparators

Authors acknowledged the challenge of finding a comparator population that could serve as an appropriate control for those receiving the intervention. Observational designs were often adopted because of the difficulty in identifying participants for a comparator group. Olsson et al. note that it would have been ideal to randomize community-dwelling older adults to usual care vs. a multidisciplinary geriatric primary care team. However, it was impractical due to the administrative burden: “there were concerns about the difficulty for nursing staff working in two care systems at the same time and for patients possibly comparing the treatment they received “[64, p. 1633]. In many observational studies, the significant baseline differences between those receiving the intervention and usual care emerged as a major threat to the studies’ validity. In an integrated intervention targeting high cost Medicare users, Mccal et al highlight that: “…because the comparison group was not based on random sampling….if the intervention had a disproportionate number of high risk, more cost increasing beneficiaries, then the [the evaluation results] could be biased against the intervention” [57, p. 143]. However, even in RCTs, the hidden differences in the context of the comparator and the intervention group could jeopardize the attribution of effect, or causality [15]. To address this potential bias, studies adopted quasi-experimental approaches to adjust for baseline differences between comparison groups. In a cost-effectiveness study of disease management programs in the Netherlands, Tsiachristas et al. note that, “[because] of the observational study design, the patients in the comparators in each disease category differed in disease severity and sociodemographic characteristics at baseline. Therefore, we used propensity score matching to reduce confounding caused by these differences” [40, p. 978].

#### Risk of contamination bias

Controlling for the potential of contamination bias emerged as a challenge in both observational and non-clustered RCT designs. Often due to feasibility issues, both usual care and the integrated care intervention were delivered in the same care setting, alongside each other. Zulman et al. suggest that in their study, clinicians in the usual care group could have observed and adopted the practices of the intensive case management program in the Veterans Affairs medical care home program [50]. In other studies, this bias was even more difficult to control when plans to spread the integrated care intervention across the regional setting were already underway. This was exemplified by a primary care-based collaborative mental health intervention in the Netherlands. The authors note that: “when the study started, about 65% of the general practitioners in the Hague were already participating in the program” [55, p. 78]. In large scale implementations of integrated care, there is a risk of diluting the true magnitude of impact, as it can prove difficult to confine patients receiving usual care from also accessing these services [63].

#### Post-hoc evaluation culture

A significant barrier was the implementation of the interventions without planning for an economic evaluation. In a study of a community-based program integrating HIV and nutrition care in Malawi and Mozambique, Bergmann et al. noted that “the project was set up without a research design for rigorous impact evaluation” [66, p. 710]. This limited data accessibility and the suitability of the outcome measures selected for the evaluation: “we would have preferred to directly estimate the effects of the [intervention] on morbidity and mortality but did not have the data to do so” [66, p. 710]. Other observational studies that were not initially planned as experimental study designs had to rely on routinely collected data to estimate the magnitude of impact. For example, claims data from widely accessible electronic information systems were used to measure the impact of a population-based accountable care organization in Germany. While this reduced the intensity of resources required for the evaluation, authors noted that their approach may have led to the underestimation of the broader impact of the intervention, such as its effect on patient and caregiver out-of-pocket spending [63].

## Discussion

### Main results

The results of economic evaluations of integrated care should be placed in the broader context of its implementation and the methodological approaches used in its evaluation. To our knowledge this is the first review that critically appraised the methodological quality of economic evaluations of integrated care interventions, against best-practice guidelines. Our review found significant differences in quality. Some studies showed poor methodological rigor, challenging conclusions on the cost-effectiveness of integrated care. Similar to Nolte and Pitchforth’s review of systematic reviews on the economic impacts of integrated care, we found wide variability across study designs, measurements of costs and outcomes, as well as analytical approaches and presentation of results [5].

Some of the key cited challenges to robust economic evaluations in our study were related to: 1) time horizon of the evaluation; 2) inadequate or lack of comparator group; 3) contamination bias due to potential exposure of those in usual care with the treatment; and 4) a post-hoc evaluation culture.

### Interpretation of findings in the context of other studies

Several reviews highlighted similar challenges as impediments to arriving to robust evidence on the economic impacts of integrated care. In a review of interventions targeting frequent users of the emergency department, Althaus et al. note that it was difficult to attribute cost savings to the interventions when a significant number of studies did not have comparators [74]. De Bruin et al. highlight that there was a large variation in the types of comparators used in their review of the impact of disease management programs on health expenditures. They found that when there were similarities in the intervention and usual care, it was difficult to observe differences. The ability to contrast between the two groups was further challenged by the poor description of usual care conditions in the majority of the studies [13].

Because integrated care can impact a broad range of costs within and beyond the healthcare system, a broader societal perspective is preferred when estimating costs [15]. In our review, the healthcare perspective dominated the economic evaluations. This could be because it requires much less time and financial resource for data collection. While there is variation in the cost perspectives suggested by national guidelines for health technology assessment, they often also recommend reporting costs from the societal perspective. This is particularly the case if the intervention has an expected impact on other sectors [75,76]. In a review of collaborative models for individuals with depression, none of the reviewed economic evaluations included costs beyond the healthcare sector [77]. A narrow scope in the cost perspective was also reported in Wong et al.’s systematic review of economic evaluations of integrated care for cardiac rehabilitation. Using the Drummond’s check list to examine the quality of economic evaluations, they found that only 9% of studies included all the relevant costs and consequences for both the intervention and comparator [18]. This narrow scope fails to incorporate the impact of integrated care on other types of care (e.g. Social Services), government departments (e.g. Justice and Education) and productivity levels in the overall economy [32,78]. An alternative would be to follow the recommendations of the specific country of origin, and to include a societal perspective as a sensitivity analysis.

### Best practice recommendations

Guidelines for health technology assessment around the world recommend adopting a life-time horizon [16,17,23]. Drummond and colleagues recommend, that in treatment of chronic diseases where benefits may have long-lasting implications, it is often necessary to extrapolate the effects and costs of the interventions being compared over a life time [20]. However, short time horizon was acknowledged as a major limitation in most studies. In our review, 81% of the studies had less than two years follow-up period. Only one study employed decision analytic modelling to extrapolate costs and outcomes estimated during the follow-up period to patient’s lifetime [49]. This approach also allows the linkage of intermediate endpoints (e.g. clinical status) to final endpoints (e.g. QALYs and mortality) [23]. The scarcity of model-based economic evaluations in integrated care maybe due to lack of health economic modelling expertise in this field of research. Another reason could be the complex nature of integrated care, with a non-linear relationship between interventions and outcomes [15]. In addition, delays in the implementation of integrated care interventions may lead the “full” treatment effect to be observed in 3–5 years after the start of implementation [79]. Decision models would not be able to overcome this limitation. Furthermore, evaluations with longer time horizons have an increased risk of the intervention eventually becoming usual care or being contaminated by other initiatives. Tsiachristas et al. suggest setting up routine monitoring of key measures as a potential strategy to measure the long-term effects of the intervention, beyond the research period [15].

Best practice recommendations emphasize the need for a comparator in economic evaluations, often in the form of current practice or variations of similar programs [15], [16,28]. Our review found that identifying the appropriate comparator can be challenging, particularly for observational studies. To ensure that the evaluation is in fact measuring the causal effect of integrated care, an appropriate control population should be chosen [16,17]. RCTs are commonly used to minimize differences between the compared populations (i.e. intervention and control arms). However, addressing the differences in observational studies may prove difficult and resource-intensive because the richness of required data and statistical expertise. Furthermore, the risk of contamination by the control group from the intervention is elevated, especially when integrated care implementation takes place in large parts of the population. As such, even cluster-RCTs may be difficult to overcome this risk if not well designed [80]. Quasi-experimental designs provide feasible alternatives in the evaluation of integrated care, when control groups are identified. An approach such as propensity-score matching could reduce observed confounding between the comparators, and is increasingly used in observational studies [81,82]. Approaches such as difference-in-differences, instrumental variables, and regression discontinuity could reduce the unobserved confounding between the comparators [15,83].

Integrated care is considered a complex intervention, amenable to being tailored to the context in which it is implemented in. Economic evaluation plays an important role in this complex and adaptive intervention, learning through feedback loops of patients and provider experiences and outcomes [15]. Our review found that economic evaluations in integrated care were often piggy backing on larger scale evaluations. In some instances, the plans for evaluation began after the intervention had concluded [66]. When this is the case, researchers may lack control over the types of outcomes measures included in the routinely collected data [15]. In a qualitative study examining stakeholder perspectives on the evaluation of chronic disease management programmes in six European countries, lack of an evaluation culture was also cited as one the main barriers towards producing sound evidence [84]. There are several potential explanations for this. Policy makers and practitioners maybe unaware of the need for, or benefits of conducting evaluations [84]. There may be a reluctance by stakeholders to support evaluations due to perceived additional administrative and financial burdens [64]. Finally, in some health care contexts, particularly in low-resource settings, there might be a lack of personnel capacity or necessary skill sets to undertake comprehensive economic evaluations [85]. Nonetheless, with the increased reliance on evidence generated from empirical studies by decision-makers, it is important for evaluators to be embedded early, from the design and planning of the integrated care programme, rather than post-implementation [15].

### Strengths and Limitations

The extensive search strategy used to capture the concepts of integrated care and economic evaluation is a major strength of this review. A second strength is that our checklist was adapted from the CHEERS and the HTA-DM quality assessment guidelines, which were developed by experts in the field for the purpose of optimizing the reporting of evaluations and health economic evaluations [17,28]. However, this review should be interpreted in the context of several limitations. Integrated care as a concept has been widely used to achieve various objectives, which explains why there remains a lack of common definition which is universally accepted [86,87,88]. While our search strategy attempted to include studies that broadly fit with in our definition, our review may have missed others. Rather than presenting the review as a compendium of all economic evaluations conducted in integrated care, we hope this provides a snapshot into the current practices in the field. Secondly, the checklists provided a guiding framework for critically reviewing the economic evaluations reported by the articles [17]. However, there was room for subjective interpretation which may have biased the scoring. We attempted to address this bias through two reviewers independently appraising the articles against the checklist, with disagreements resolved through reaching consensus.

### Implications for research, policy and practice

Economic evaluation cannot be viewed as separate from programme evaluation. Rather, it needs to become an integral part of any evaluation effort [15]. Given the wide implementation of integrated care as a viable approach to tackling complex health and social needs, it is necessary for researchers to step up their efforts in understanding how exactly integrated care works and which transferable lessons can be drawn [88]. This necessitates a multidisciplinary approach in research, where economic evaluation forms a crucial part of a mixed methods approach. Recent developments in applying realist evaluation and multi-criteria decision analysis (MCDA) as part of implementation research in Europe and Australia show promising results [89,90]. However, they also highlight the complexity and resource intensity necessary to evaluate integrated care. For policy makers to create enabling conditions, researchers also need to become more engaged in the promotion of an evaluation culture. Routinely reporting on short-, medium- and long-term outcomes may help foster a better understanding among policy makers on adequate time horizons necessary for evaluation. Policy makers also need to provide financial and regulatory frameworks for research to become an integral part of designing integrated care initiative, similar to the Innovation Fund in Germany. More importantly, evaluation needs to be viewed as an integral part of integrated care implementation, as a powerful tool to promote cultural change [88]. This is where practice needs to accept that (economic) evaluation is not a means to punish or cut resources, but can be used to support quality improvement and system change. In a complex environment, regular on-going feedback and monitoring are prerequisites to reaching better outcomes [88]. Given the continuing resource restrictions, alongside the inadequate and ineffective use of resources in health and social care, economic evaluation should be high on any priority list, in research, policy and practice.

## Conclusion

Arguably a key challenge to the evaluation of integrated care lies in the complexity of the intervention and the short-term evaluation period. This can make it significantly more difficult to perform rigorous economic evaluations; especially when health economists are not involved in the study design. However, methodological gaps in economic evaluations may be more straightforward to address than cultural barriers to evaluation [20]. Therefore, it is important for evaluators to use best-practice standards when planning and conducting economic evaluations. Work by authors such as Drummond et al. has sought to specify the essential components of good economic evaluations in health care and to develop checklists that can help researchers and practitioners critically appraise the methodological quality of studies [17,19,20]. To build a reliable evidence base for decision-makers and practitioners utilizing evidence from integrated care studies, it is important to determine the mechanisms that influence the economic impacts of integrated care. This will require designing studies that can reliably answer: is this intervention cost-effective, using which resources, in which settings and for whom? [15] With the increased interest in integrated care from policy makers, the reporting of economic evaluations should be further standardized to allow transferability and transparency [20]. Thorough and reliable economic evaluation should be an integral part of informing the decision-making of integrated care implementation.

## Additional File

The additional file for this article can be found as follows:

10.5334/ijic.4675.s1 Appendix 1. Search terms.
