# Supplementary material for: Methodological Quality of Economic Evaluations in Integrated Care: Evidence from a Systematic Review
Source: Int J Integr Care. 2019 Sep 9;19(3):17. doi: 10.5334/ijic.4675 (PMC6743034; doi:10.5334/ijic.4675)
Supplement: Appendix 1. — Search terms. [file ijic-19-3-4675-s1.pdf]

APPENDIX 1: Search terms

PUBMED/SCOPUS/EMBASE/MEDLINE/Ovid/CINAHL/NHS EED + Dare + Google searches:

((‘integrated* care’) OR (‘integrated* delivery) OR (‘integrated* system’) OR ('transmural care') OR (('chains of care') OR ('collaborative* care') OR ('cooperative* care') OR ('coordinated* care') OR ('continuous* care') OR ('systems* care') OR ('intersectoral* care') OR ('interdisciplinary care’)* OR ('cross sectoral* care') OR ('linked care') OR ('seamless care') OR ('shared care') OR ('transitional* care') (‘multidisciplinary care’) OR (‘care linkages’) OR (‘care pathway’) OR (‘seamless care’) OR (‘shared care’) OR (’virtual integration*’) OR (‘horizontal integration*’) OR (‘functional integration*’) OR (‘normative integration*’) OR (‘collaborative* care’) OR (‘cooperative* care’) OR (‘information* continuity’) OR (‘interpersonal continuity’) OR (‘management continuity’) OR (‘managed care’ OR (‘disease management’) OR (‘case management’) OR (‘case coordination’))

AND (('cost utility*') OR ('cost effectiveness*') OR ('cost benefit') OR ('economic evaluation') OR ('cost impact') OR ('economic impact') OR ('cost savings*') OR ('cost consequence*') OR ('cost evaluation') OR ('economic appraisal') OR (‘cost comparison') OR (‘decision modelling’))
